# Radiation oncology as a complex adaptive system

**DOI:** 10.3389/fonc.2025.1686835

**Published:** 2026-01-16

**Authors:** Mohammad Bakhtiari

**Affiliations:** Department of Radiation Oncology, WellSpan Health, Chambersburg, PA, United States

**Keywords:** adaptive, complex system, complexity, radiation oncology, system thinking

## Abstract

**Background:**

Radiation oncology (RO) is increasingly recognized as a complex system characterized by the intricate interplay of professionals, patients, technology, and environmental factors. However, systematic methods to assess the interconnectivity in RO remain limited, leading to potential oversimplifications of this multifaceted field. This study quantitatively assesses evolving complexity and nonlinearity in radiation oncology using system-thinking tools. The goal is to create a measurable framework to guide adaptive management in clinical practice.

**Methods:**

Using Shannon Entropy, we analyze the evolving complexity in RO. Dynamic system simulations, including adapted predator-prey models and the SimPy discrete-event simulation library, are utilized to study nonlinearity and interactions within RO. Process mining with event log data assesses the conformance of RO processes, while social network analysis explores self-organization among RO actors.

**Results:**

Our findings reveal a marked increase in the complexity and nonlinearity of RO. Simulations and process mining demonstrate emergent behavior, self-organization, and adaptability within the system.

**Conclusions:**

The inherent nonlinearity, complexity, emergence, adaptability, and self-organization in RO systems validate the view of RO as a complex system. This insight calls for a shift towards ‘system thinking’ in managing, leading, and operating within the RO field. This approach will better accommodate the dynamic, interconnected nature of RO, ensuring more effective and adaptable healthcare outcomes.

## 1 Introduction

The Institute of Medicine’s (IOM) groundbreaking report “Crossing the Quality Chasm” (1) and “To err is human” (2) offer a crucial lens through which to understand the inherent complexities of healthcare systems. Emphasizing the “complex system paradigm,” the reports demonstrate the intricate interplay of factors, variables, and stakeholder interactions that shape healthcare outcomes. Rather than adopting a reductive approach that isolates individual elements, the reports advocate for “systems thinking,” a holistic method that grasps the web of relations within healthcare (3, 4). The report emphasizes systems thinking, providing a clear understanding of current challenges and outlining a progressive path forward. This approach utilizes the system’s complexities to enhance quality improvements and patient safety. System thinking is also frequently emphasized in radiation oncology reports and frameworks (5, 6). Complex systems are often unpredictable, so in this work, we aim to demonstrate the validity of that assumption in the context of Radiation oncology (RO). We hesitate to quantify results, which may seem unconventional for research. However, it’s important to note that thinking systems require different approaches.

Systems are either simple, complicated, or complex (7). The distinction between complicated and complex systems is essential, as these two systems have distinctive characteristics and behaviors. Simple and complicated systems are characterized by linear and predictable behavior. In contrast, complex systems exhibit non-linear and emergent behavior that is often difficult to predict or control. Understanding the differences between these three types of systems can help us analyze better, design, and improve the systems we interact with. Linear accelerators are among either simple or complicated systems regardless of their sophistication, as they run linearly and predictably, and issues are usually traced to a specific cause-and-effect relationship (7). In case of a malfunction, resolving the problem may involve following a predetermined checklist or enlisting the help of a specialized expert (service engineer). On the other hand, RO, like any other healthcare setting (8), comprises a team, including patients, radiation oncologists, medical physicists, medical dosimetrists, radiation therapists, and other professionals such as Information Technologists (IT), nurses, dietitians, social workers, and front desk (5). The events in RO are unpredictable due to the interconnectedness of different elements, where cause and effect are interchangeable. The team members constantly interact with technological elements known as artifacts, such as computers and linear accelerators, to perform their tasks. This interaction places RO in a sociotechnical category (9, 10). As technology advances, human beings cannot keep pace with understanding and controlling it due to the slow pace of human evolution. It is also essential to understand that RO interacts with various environmental factors, such as government regulations, politics, the economy, weather, traffic, etc. These properties make RO a complex sociotechnical system (5). Lastly, A complex system that can adapt or learn in response to its environment or interactions is called a Complex Adaptive System (CAS) (11). Feedback mechanisms are crucial in driving adaptation, which enables a system to self-organize and adjust its behavior over time. In the case of a complex system like resource optimization, which is highly influenced by external factors such as weather, politics, traffic, and others, it becomes necessary to adapt to environmental changes to maintain equilibrium within the system.

Systems thinking, a robust framework, views healthcare as a complex ecosystem. It emphasizes the interconnectedness and interdependence of all elements, from patients and providers to treatment technologies and healthcare infrastructure. This holistic approach contrasts with traditional, reductionist views focusing on individual components. For example, a simple change in treatment plan can ripple through the system, affecting care delivery and patient outcomes. Although numerous publications acknowledge the complexity of healthcare systems and radiation oncology (5, 6), it is rare to find a source in the literature that provides a rigorous quantitative framework to prove this complexity. The motivation for this study is to quantitatively assess the evolving complexity and nonlinearity in RO by applying system-thinking tools to develop a measurable framework for adaptive risk management. While it is well-established that healthcare systems function as complex adaptive systems (CAS), many RO departments continue to rely on traditional, linear approaches for problem-solving and management. This mismatch between the field’s rapid technological growth, interdisciplinary collaboration, and dynamic workflows on one hand, and relatively static management practices on the other can hinder effective adaptation and compromise safety. This gap has become more pronounced with the increasing adoption of adaptive radiation therapy, where real-time imaging, plan modification, and decision-making introduce additional layers of interdependence and nonlinearity (12).

Recent advances in Artificial Intelligence (AI) have begun to transform radiation oncology by automating aspects of treatment planning and quality assurance. However, AI integration also introduces additional complexity, leading to unpredictable incidents that standard risk assessments may fail to anticipate. By providing empirical measurements of complexity and nonlinearity in RO, our study aims not only to characterize RO as a CAS but also to offer actionable insights that can guide adaptive strategies and improve clinical outcomes. Recent multidisciplinary guidance, such as the ASTRO White Paper on adaptive radiation therapy, has emphasized that these evolving, patient-specific workflows substantially increase system complexity and require coordinated, team-based quality and safety strategies rather than static protocol-driven controls (13).

Recent literature has increasingly emphasized the need for adaptive safety and governance frameworks to manage the growing complexity introduced by advanced technologies, including AI-driven planning, automation, and decision support systems in radiation oncology (14, 15). These efforts highlight important challenges related to validation, oversight, and unintended consequences in dynamic clinical environments. However, most existing approaches focus on specific tools or failure modes rather than quantitatively characterizing the underlying system-level complexity in which these technologies operate. The present study addresses this gap by providing a multi-method, quantitative demonstration of complexity, nonlinearity, emergence, and self-organization in radiation oncology, establishing a systems-level foundation for contemporary risk management and AI governance.

The properties of CAS can vary depending on the specific context and application. There is no definitive list of properties that define CAS (9, 16, 17). In this manuscript, we will introduce some properties of CAS in section 1. Then, we will utilize methods to investigate these properties in section 2. The results will be presented in section 3, while the discussions and conclusion will be covered in sections 4 and 5.

### 1.1 Spatiotemporal dynamics

**Figure 1:**
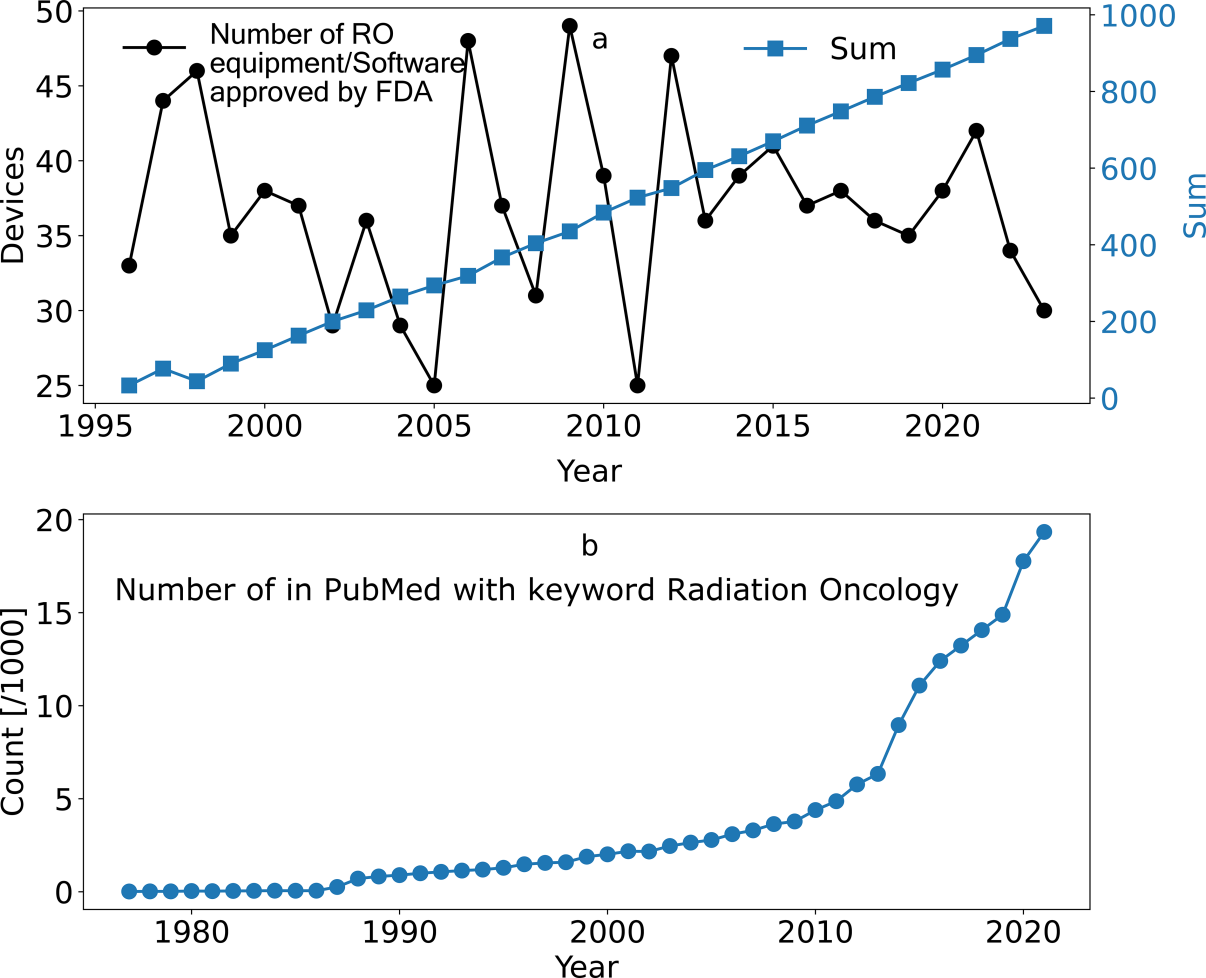
**(A)** number of radiation oncology equipment/software systems approved by FDA, **(B)** number of in PubMed with keyword “Radiation Oncology” in various years.

The complexity is space and time-dependent. **Figure 1A** shows the data from the Food and Drug Administration (FDA) on approved radiation oncology devices and software packages each year for the past several decades (18). According to the World Health Organization report titled “Radiotherapy Risk”, this is important because 45% of the incidents happened when new systems or tools were introduced (19). Another example of the spatiotemporal dynamic of the oncology field is shown in **Figure 1B**, indicating that the number of publications on radiation oncology in PubMed is exponentially increasing. Radiation oncology is also shaped by spatial variability, as shown in several works (20, 21). In the context of radiation oncology, “spatial variability” refers to differences due to geographical and organizational factors. This includes variations across different locations, such as departments within the same hospital, different hospitals, regions, or even countries. These variations can affect everything from the availability of technology and expertise to the methods of treatment and patient management practices. The complexity of Radiotherapy Oncology, dictated by both temporal and spatial factors, underscores a critical reality: a solution effective in one specific time or location may not be suitable or could even pose challenges in a different time or space. This highlights the need for adaptable and context-sensitive radiation therapy approaches.

### 1.2 Nonlinearity

Nonlinearity refers to the idea that a change in the input of a complex system can lead to an un-proportional shift in the output (22). Nonlinearity in RO results in the system’s unpredictable and often chaotic nature. The RO teams are composed of multiple individuals with distinct roles, responsibilities, and decision-making processes, which can result in unexpected and nonlinear outcomes. Individuals are not interchangeable and can be biased. These biases also influence nonlinearity and chaotic behavior. Feedback loops are critical features of complex systems, where the system’s behavior is influenced by the interactions and relationships among its components. In a feedback loop, the output of a process is fed back into the system as an input, which then influences the system’s behavior. This dynamic can cause the system to become both influenced by and an influencer of its outputs, creating an ongoing cycle where effects and causes intertwine (23). Feedback loops allow the system to learn from past experiences and adjust its behavior accordingly (23). This can include positive feedback, which reinforces behavior that leads to success, and negative feedback, which corrects behavior that leads to failure. Several feedback loops can interact and create more nonlinear and unpredictable dynamics. These feedback loops can be found in a wide range of complex systems, from ecosystems and economies to social networks and technological systems, and understanding them is essential for effective management, decision-making, and investigating incidents (23). Other terms used for negative loops in different contexts are balancing loops, homeostatic, or stabilizing loops (24). Positive loops are also called reinforcing or amplifying loops (24). In RO, due to nonlinearity, unpredictable safety and quality issues may arise (25).

### 1.3 Emergence

Emergence, as a fundamental property of complex systems in healthcare, signifies the spontaneous creation of order and novel patterns from the local interactions among system components without a central command (8, 24). This concept is particularly pertinent to RO, where diverse elements interact in multifaceted ways to produce outcomes that are often unpredictable from the behavior of individual components. In the context of RO, emergence can be observed in the way collaborative efforts among healthcare professionals lead to improved patient outcomes that could not be achieved by individuals working in isolation. This synergy—from coordinated efforts, knowledge sharing, and decision-making processes—leads to emergent properties like quality and safety in patient care (5, 11). The emergent property in RO is the ultimate treatment outcome, safety, and quality, which results from the interplay between the expertise of the team members, the precision and reliability of the technology used, and the adherence to protocols and safety standards. That’s how the IOM reports consider safety and quality as system properties rather than static attributes (5). In essence, emergence in healthcare recognizes that the whole is greater than the sum of its parts. It’s about seeing beyond individual actions and understanding how those actions combine and intersect to produce outcomes (26).

### 1.4 Self-organization

Self-organization is the process by which a system of components or agents forms a coherent structure or pattern through interactions without external direction or control. Self-organizing is a property of a CAS (9). An open system exchanges matter, energy, and information with the environment. The process of self-organization allows the system to adapt and evolve in response to changes in its environment and can lead to the emergence of new properties and behaviors. Self-organization can be referred to as being “away from the resting state.” (27). These systems exhibit high autonomy and adapt to changing environmental conditions, making them resilient and robust (28). RO systems are examples of CAS maintaining homeostasis (29, 30). In Ref (26), Damasio discusses how homeostasis is not just a biological necessity but also a foundational aspect of consciousness and behavior in complex systems.

Despite the robust theoretical foundation provided by systems thinking and the well-documented properties of complex adaptive systems (CAS), many RO departments continue to employ traditional, linear management and risk assessment methods. This disconnect between the dynamic, non-linear nature of modern RO and the static, compartmentalized approaches in practice can lead to suboptimal adaptation and compromise patient safety. In response to this gap, the present study aims to quantitatively assess the evolving complexity and nonlinearity in RO by employing system-thinking tools. Our objective is to develop a measurable framework for adaptive risk management that can guide improvements in clinical outcomes and operational efficiency.

## 2 Methods

### 2.1 Spatiotemporal dynamics

We utilize Shannon Entropy to demonstrate the complexity (31). Shannon entropy is a measure of the uncertainty or disorder of a system. In a network, Shannon entropy can be calculated by determining the probability distribution of the different degrees of the nodes or the number of connections each node has to other nodes in the network. The probability of each degree is then used to calculate the entropy using the formula: H=−∑​p(k)log(p(k)), where *H* is the entropy, *p(k)* is the probability of a node that has degree *k* and the sum is above all degrees in the network. To compute *p*(*k*), we first determine the degree of each node within the network. We then count the number of nodes *n*_*k*_ that have each specific degree *k*. The probability *p*(*k*) for each degree *kk* is calculated as *n*_*k*_ divided by the total number of nodes in the network. For this analysis, we assume each node (e.g., person, device, or sub-process) contributes equally, and we derive p(k)p(k)p(k) from the observed degree distribution. We recognize this may not capture external factors such as workforce growth, economic constraints, or the unique impact of particular technologies, and we consider this a limitation of the current approach. Nevertheless, Shannon Entropy provides a useful baseline for comparing network heterogeneity over time. This approach allows us to assess the randomness and distribution of connections within the network, as entropy increases when the network configuration becomes more heterogeneous. Finally, the entropy value is summed over all degrees, providing a measure that reflects the overall complexity of the network structure. This metric is crucial for understanding how network connectivity influences system behavior, offering insights into the underlying patterns of interaction within the network. Because some innovations—particularly transformative technologies such as Volumetric Modulated Arc Therapy (VMAT)—may outsize the complexity contribution of smaller incremental changes, treating all components with equal weight may underestimate the system’s true complexity. We therefore consider our present entropy values to be conservative estimates, implying that actual complexity is at least as high as, if not higher than, these values suggest. This finding further supports our central objective of demonstrating the growth and significance of complexity in modern radiation oncology.

A network with a high Shannon entropy is more random or disordered, as the probability distribution of the node degrees is more evenly distributed. A network with a low Shannon entropy is considered more ordered, as the probability distribution of the node degrees is more skewed towards certain degrees. For example, gas molecules with a high degree of freedom have remarkably high entropy. In contrast, a solid crystal structure has zero entropy as the molecules are bound to a particular structure and order. This analogy is also valid for a department with no order or chaos or a department with very tight order in place. Shannon entropy can be used to analyze the structure of complex networks and compare the randomness or disorder of different networks. It can also be used to analyze the changes in network structure over time and to identify patterns of node interactions and connections.

For this part of the study, we used empirical data shown in **Figures 1A, B**, as inputs to calculate the Shannon entropy for a team of eight, in different years to investigate the change in Complexity.

### 2.2 Nonlinearity

Dynamic system simulations are utilized to model the interactions between system elements. First, we apply a modified predator-prey model (32) to analyze the dynamics between the workload (number of plans, *Np*) and the pressure on dosimetrists (*P*), and their impact on performance in a radiation oncology setting. This is a deterministic approach using differential equations: $\dot{N_{p}}=aN_{p}-bN_{p}P$ and $\dot{P}=-cP+dbN_{p}P$, where: *a* is the rate of plan generation, *b* represents the effect of the number of plans on increasing pressure on dosimetrists, *c* is the natural reduction rate of pressure when plans are not being processed, and *d* signifies how pressure increases based on the number of plans. The performance metric is defined as $Performance=e\times b\times N_{p\;}\times P$. Here, *e* is the performance coefficient. We often observe calendar effects on the workload.

Second, we employed a probabilistic method. SimPy is a process-based discrete-event simulation library for Python (33) that models systems by describing the interactions between various entities or components as a series of events that occur over time. The simulation models the relationship between pressure, performance, errors, workload, and patient satisfaction.

Dynamic system simulations are vital for dissecting the complex dynamics of workload, pressure, and performance in radiation oncology. These simulations permit the manipulation of variables (a, b, c, and d) in scenarios unattainable in real-world settings due to ethical, logistical, and financial constraints, uncovering nuanced effects on performance through a controlled environment. This method facilitates the exploration of theoretical models, highlighting mechanisms and predicting outcomes in complex systems where establishing empirical causal relationships is challenging. It allows for the identification of critical thresholds and tipping points. It is a precursor to empirical validation by generating specific, testable hypotheses about the interactions between workload (Np) and pressure (P). The deterministic approach of differential equations offers a clear, replicable description of the system’s dynamics, laying the groundwork for future empirical studies. Despite empirical data being the gold standard, the unique challenges in this field make simulations not only justified but essential, providing a comprehensive understanding and informing future empirical research. Hence, simulation is crucial for advancing knowledge in this area.

### 2.3 Emergence

Process mining (34) is a field of study that uses data mining techniques to analyze and understand business processes. The goal of process mining is to extract knowledge from event logs, which are records of the activities that take place within a business process. Among the different features of process mining is Conformance checking (35). This technique compares the actual process behavior to the predefined process model. It detects deviations, providing a fitness metric that measures how well the observed behavior conforms to the expected behavior. In process mining, “fitness” indicates the degree to which a process model—whether discovered or designed—corresponds with the actual execution of a business process, as recorded in an event log. This concept evaluates the model’s precision and capacity to replicate the observed behaviors.

#### 2.1.1 Data extraction and preparation

We obtained our event log data from the Electronic Medical Record (EMR) system, focusing on completed patient cases that spanned the entire RO workflow—from simulation (CT Sim) to end of treatment. All data was completely anonymized; only the timestamps for the treatment planning activity were used. To ensure consistency and reliability, we performed the following data preparation steps:

##### 2.1.1.1 Selection of relevant events

We filtered event logs to include only those case IDs (patients) who had undergone the full clinical workflow. This excluded partial treatments or any patients who did not complete the full course of therapy (e.g., canceled cases).

##### 2.1.1.2 Data cleaning and validation

Timestamp Consistency: We removed entries with missing or obviously incorrect timestamps (e.g., future dates beyond the end of data collection). Inconsistent or out-of-sequence timestamps were flagged and either corrected (where possible) or omitted.

Duplicate Entries: Duplicate logs with the same timestamp and activity label were consolidated to avoid over-counting.

##### 2.1.1.3 Normalization and labeling

Standard Activity Naming: To avoid confusion, we standardized activity labels (e.g., “CT-Sim,” “Planning,” “QA Check,” “Treatment”) so that pm4py could recognize each distinct step.

Unified Case Identifiers: We ensured that each patient’s identifier was consistently labeled across all events, preventing split or merged traces due to typos or varied formatting in the EMR.

##### 2.1.1.4 Event log filtering

After cleaning, we rechecked the data for continuity (i.e., whether a trace contained all expected key stages from CT Sim to treatment). Any cases missing critical activities were discarded to maintain a coherent sequence of clinical events.

The final dataset thus reflects completed, chronologically valid event traces from simulation to end of treatment, ensuring that conformance checking accurately captures the entire care pathway—a code snippet included as a supplement for data processing.

#### 2.1.2 Process mining software

We used PM4Py (36) for our process mining analysis, which offers a variety of process discovery and conformance checking algorithms. Concretely:

##### 2.1.2.1 Process discovery

We extracted process models from the cleaned event log to identify the most frequent pathways patients follow during treatment.

##### 2.1.2.2 Conformance checking

We compared the observed traces against a predefined reference model of the ideal workflow, computing a fitness metric that indicates how closely practices align with the intended protocol.

By integrating these steps and tools, we aimed to reveal hidden workflow patterns, detect bottlenecks or deviations, and generate insights into how to better coordinate the myriad activities within a radiation oncology department. This structured approach to data preparation and validation helps ensure that our process mining results accurately reflect real-world processes rather than data inconsistencies or incomplete logs.

### 2.4 Self-organizing

Social network analysis uses process mining, discussed in section 2.3, to identify the interactions between the actors involved in the process and their roles. In some parts of the process mining technique, we analyzed the interactions between actors involved in patient treatment planning. These interactions can be represented as sequences of events that capture the actions performed by the actors, the resources used, and the time and context of the interactions (37).

## 3 Results

### 3.1 Spatiotemporal dynamics

**Figure 2:**
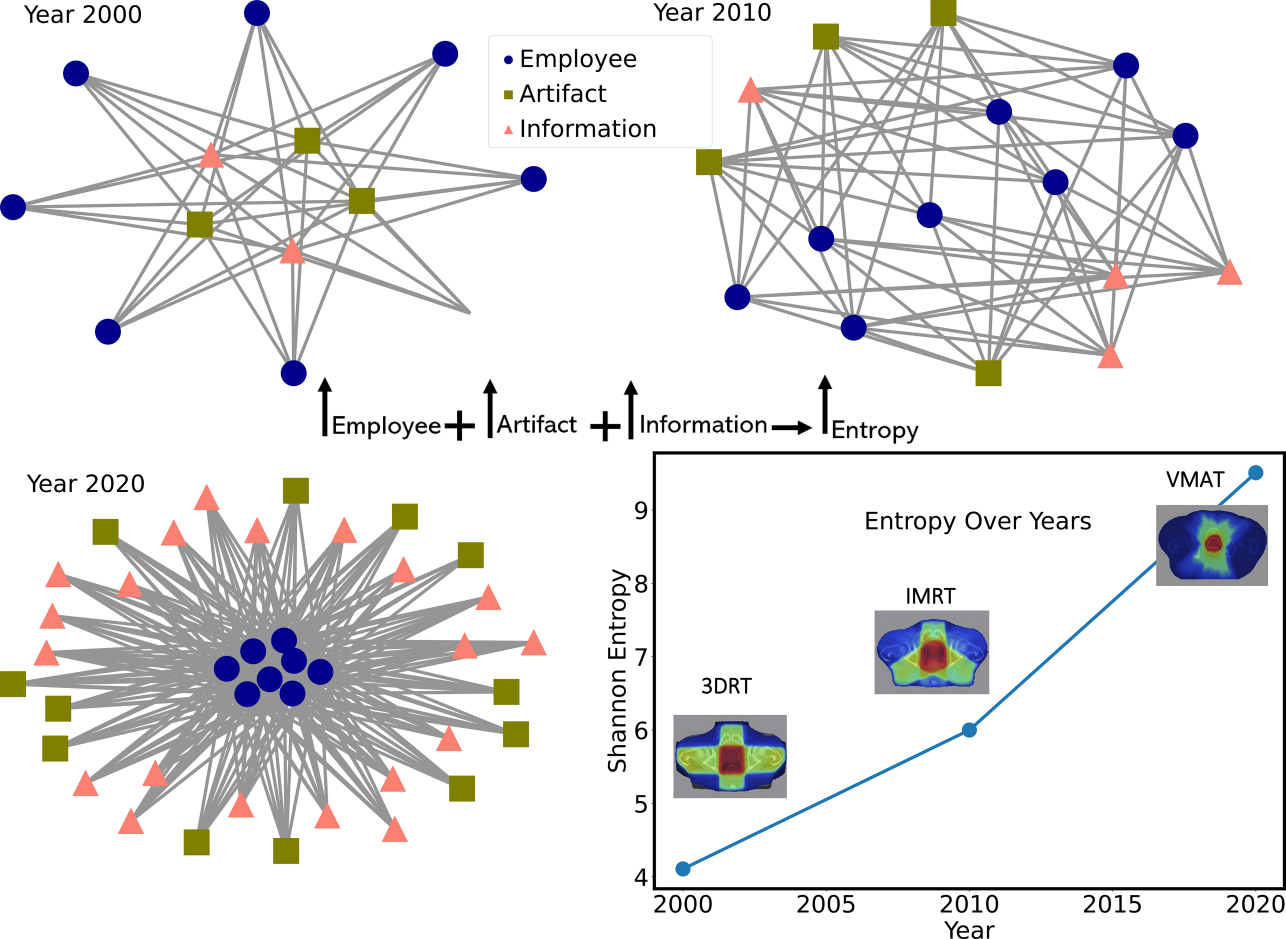
Hypothetical representation of a radiation oncology department with the same number of team members across multiple time points. “Artifacts” (e.g., linear accelerators, treatment planning software) represent technological or physical additions to the system, while “information” (e.g., publications, new protocols, medical data) denotes evolving knowledge, guidelines, and processes. This illustrative graph does not reflect actual clinical data but instead demonstrates how adding more artifacts and information over time can increase the system’s overall complexity. The purpose is to highlight how each new component—whether a device, software, or guideline—can expand the network of interactions within a radiation oncology team. For comparison, a hypothetical “non-complex” system (simple or complicated) with a steady, non-interconnected, unchanging environment would show a much lower (or near-zero) increase in artifacts and information, corresponding to a more linear and predictable state with little to no rise in entropy.

Building upon the method outlined in the Methods section 1.2, we calculated the Shannon entropy for three decades, considering the number of AAPM reports and the constant increase in the number of machines/software systems, as illustrated in **Figure 1**. This calculation supplies an estimate of the evolving complexity within the field of radiation oncology. Shannon Entropy increased approximately 4-fold over 20 years, indicating a significant rise in system complexity. In contrast a simple (non-complex) system, the cause-and-effect relationships are well understood and stable, so the system can be managed effectively with an existing set of rules or procedures, eliminating the need for continuous information updates. The graphical representation of the networks in different years is shown in **Figure 2**. We acknowledge that not all devices or publications contribute equally to complexity; some have far more significant impact than others, such as practice-changing publications or revolutionary devices, which are more widely adopted. These simple calculations are only a demonstration. Increasing complexity does not inherently imply disruptive chaos. Instead, it reflects the system’s ability to evolve and adapt to new challenges, ultimately leading to advancements in patient care. The analysis reveals a notable increase in complexity (Shannon Entropy) over the observed period. As depicted in **Figure 2**, this growth exhibits an exponential trend. This observation is significant as it underscores the rapidly evolving nature of radiation oncology, driven by technological advancements and increasing data volumes. As shown in **Figure 2**, in 2000, during the 3D Conformal Radiotherapy (3D-CRT) era, a human could calculate Monitor Units (MUs) using a pen and paper. By 2010, with the advent of Intensity-Modulated Radiotherapy (IMRT), MU calculations became significantly harder to perform manually. In 2020, the introduction of Volumetric Modulated Arc Therapy (VMAT) made manual MU calculations for each segment virtually impossible for a human. Consequently, decentralization becomes a critical necessity. Although it is impossible to control the system entirely, monitoring and feedback through minor errors and iterative adjustments enabled by incident learning systems allow the system to be steered and navigate its complexity. This exponential increase in complexity demonstrates that while the system has become more challenging to manage intellectually by solo individuals, it has simultaneously improved clinical outcomes, reduced patient suffering, and enhanced treatment reliability and safety. Such a trend in complexity has profound implications for clinical practice and research in radiation oncology. It emphasizes the need for adaptive systems and teamwork to manage this complexity. Adaptive systems, teamwork, and distributed decision-making are essential to manage this increasing complexity effectively. In a non-complex system, predictability results in a low (or near-zero) entropy value, reflecting its stable and linear nature.

### 3.2 Nonlinearity

**Figure 3:**
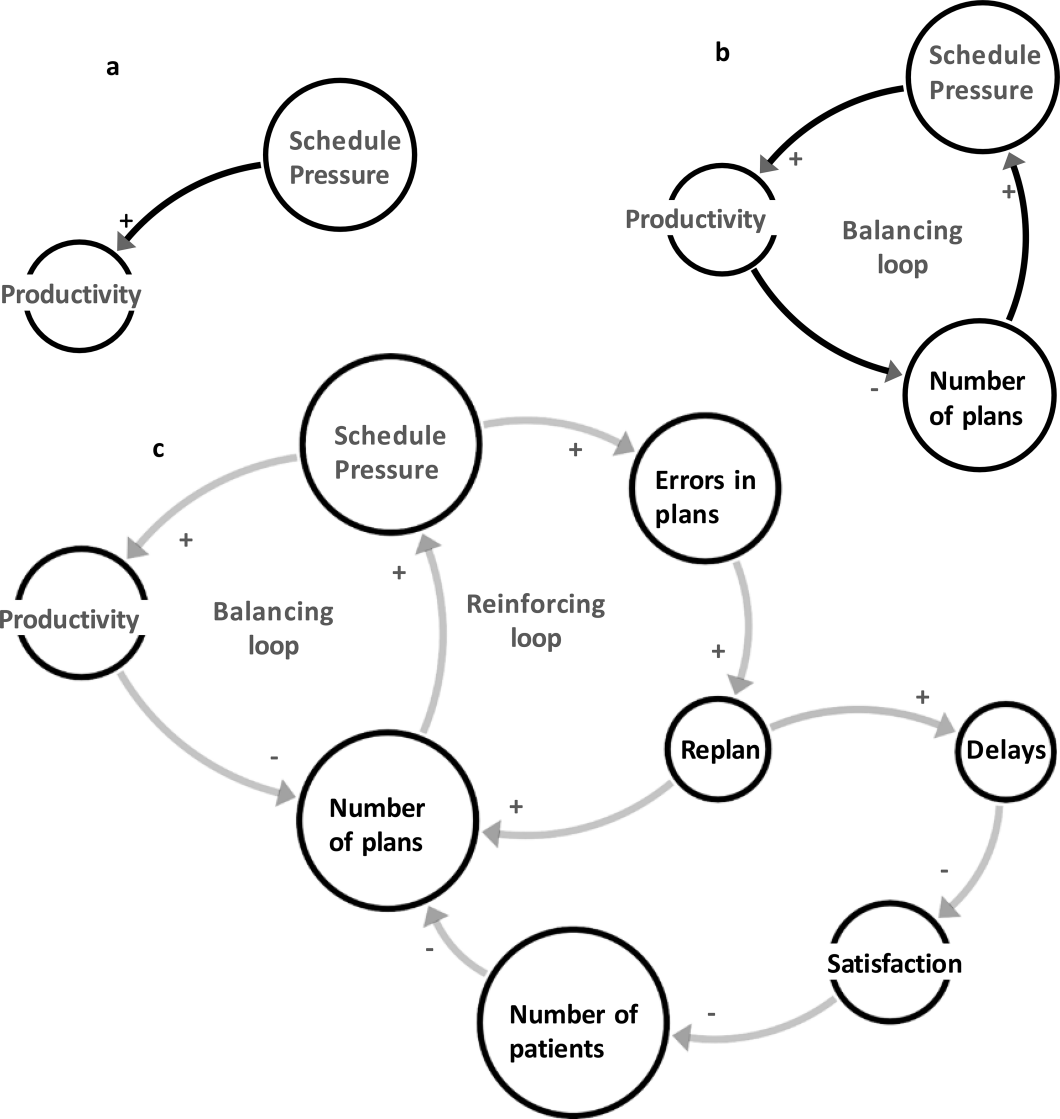
Three ways of presenting a problem/system. **(A)** Linear thinking, representing a “non-complex” or “simple/complicated” scenario with purely linear behavior and no emergent feedback loops. **(B)** simple nonlinear thinking, **(C)** more detailed nonlinear dynamics.

**Figure 4:**
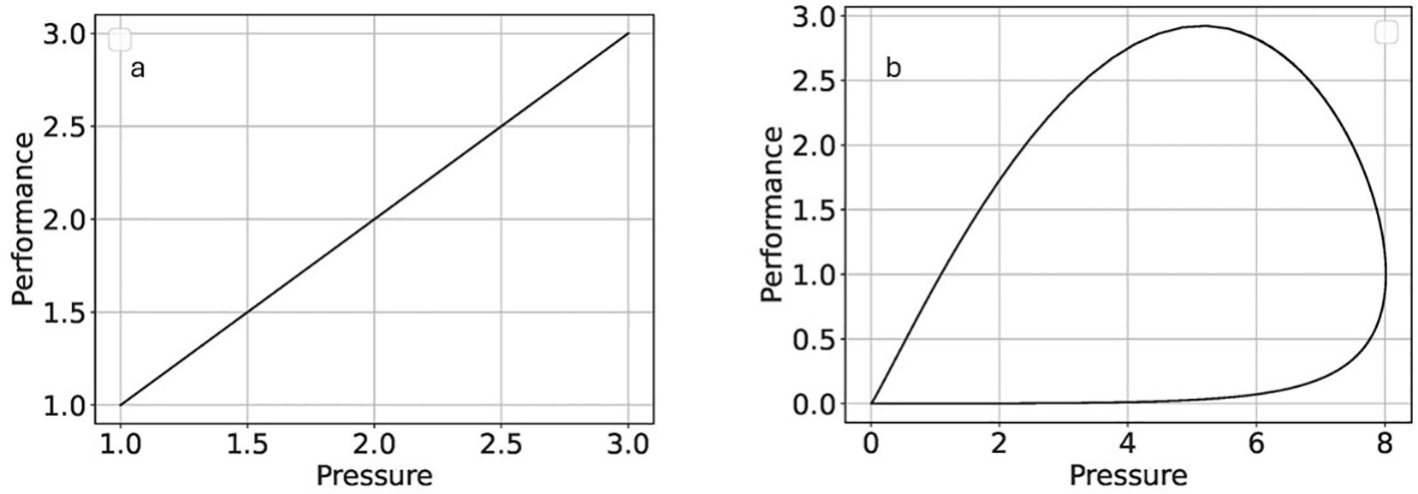
The simulations of systems in **Figures 3A, B**; linear **(a)** and nonlinear **(b)** thinking.

In **Figure 3A**, we have shown traditional linear thinking in a department. To increase performance, one needs to pressure the employee (**Figure 4A**). However, simplified nonlinear thinking, as shown in **Figure 3B**, forms a close loop as a balancing loop. More pressure on the dosimetrist increases the performance, consequently reducing the number of plans that reduce the pressure on the dosimetrist. This model provides a simplified representation of the interactions in the treatment planning process. Solving the differential equation given in Section 2.2, these relationships are nonlinear (**Figure 4B**). Although this predator-prey model demonstrates the intrinsic feedback loops within a complex adaptive system, it does not explicitly account for workforce growth, financial constraints, or regulatory changes. These external factors can significantly shift the balance of resource availability and demand, further altering system behavior. Future refinements could introduce parameters for workforce capacity, budgetary fluctuations, and policy mandates to improve real-world applicability.

System thinking can be deepened to model the intricate dynamics between pressure, performance, errors, work remaining, and patient satisfaction in a treatment planning context (See **Figure 3C**). As the pressure on the dosimetrists increases, their performance improves, leading to a reduction in the work remaining. However, the increased pressure also leads to a higher probability of errors, causing the need for re-planning and improving the overall work. Furthermore, the errors result in delays in patient treatment, adversely affecting patient satisfaction. This reduced patient satisfaction, in turn, leads to a decrease in the number of new patients seeking treatment at the center, consequently impacting the work remaining (**Figure 3**). The parameters we used in this simulation are initial pressure = 1, initial performance = 1, initial patient satisfaction = 1, error rate = 0.1, simulation time = 20, and time step = 1. We adopt the normalized initial value=1 for each parameter to highlight relative changes.

**Figure 5:**
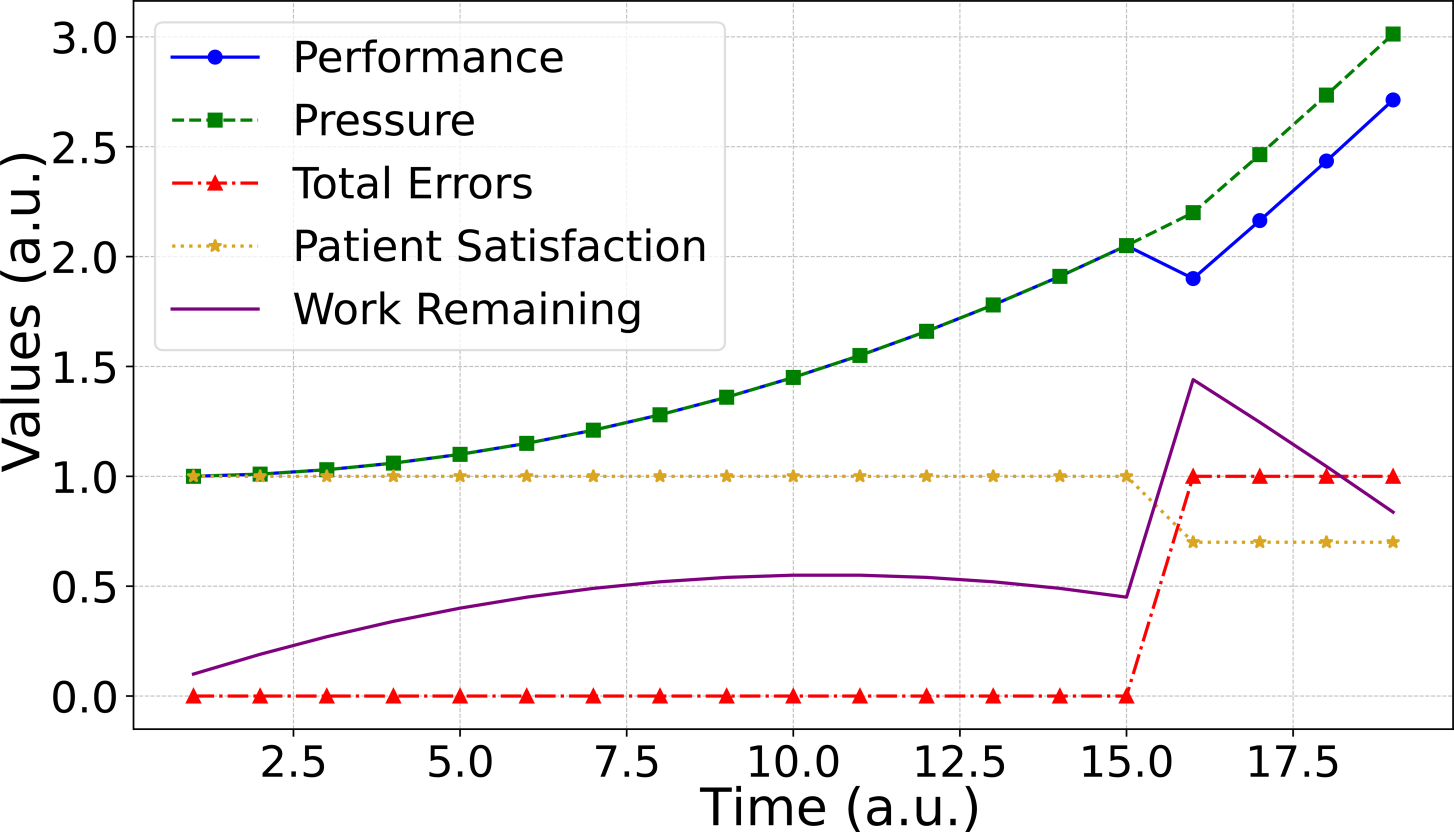
Simulating a holistic view of a system (**Figure 3C**).

The analysis of the simulation output highlights the trade-offs between applying pressure to improve performance and the unintended consequences of increased errors and reduced patient satisfaction (**Figure 5**). Note that when pressure rises beyond a specific limit in real-world systems, the employee quits, or the errors become harmful. While this model focuses on a specific sub-system, real-world radiation oncology systems involve significantly greater complexity, with thousands of agents and feedback loops shaping outcomes. Factors such as workforce growth, resource availability, financial constraints, and other external dynamics play essential roles. Future models should aim to capture such details, enabling more actionable insights and recommendations. These scenarios also reflect the inherent unpredictability of complex systems, where slight variations in initial conditions, staffing levels, or error probabilities can produce vastly different outcomes. Consequently, while our model highlights crucial feedback loops in treatment planning, it should be interpreted as a demonstration of complexity rather than a fully reproducible or universal solution. A configuration that works effectively under one set of conditions may lead to unintended consequences under another, indicating the emergent and context-dependent nature of complex adaptive systems.

### 3.3 Emergence

**Figure 6:**
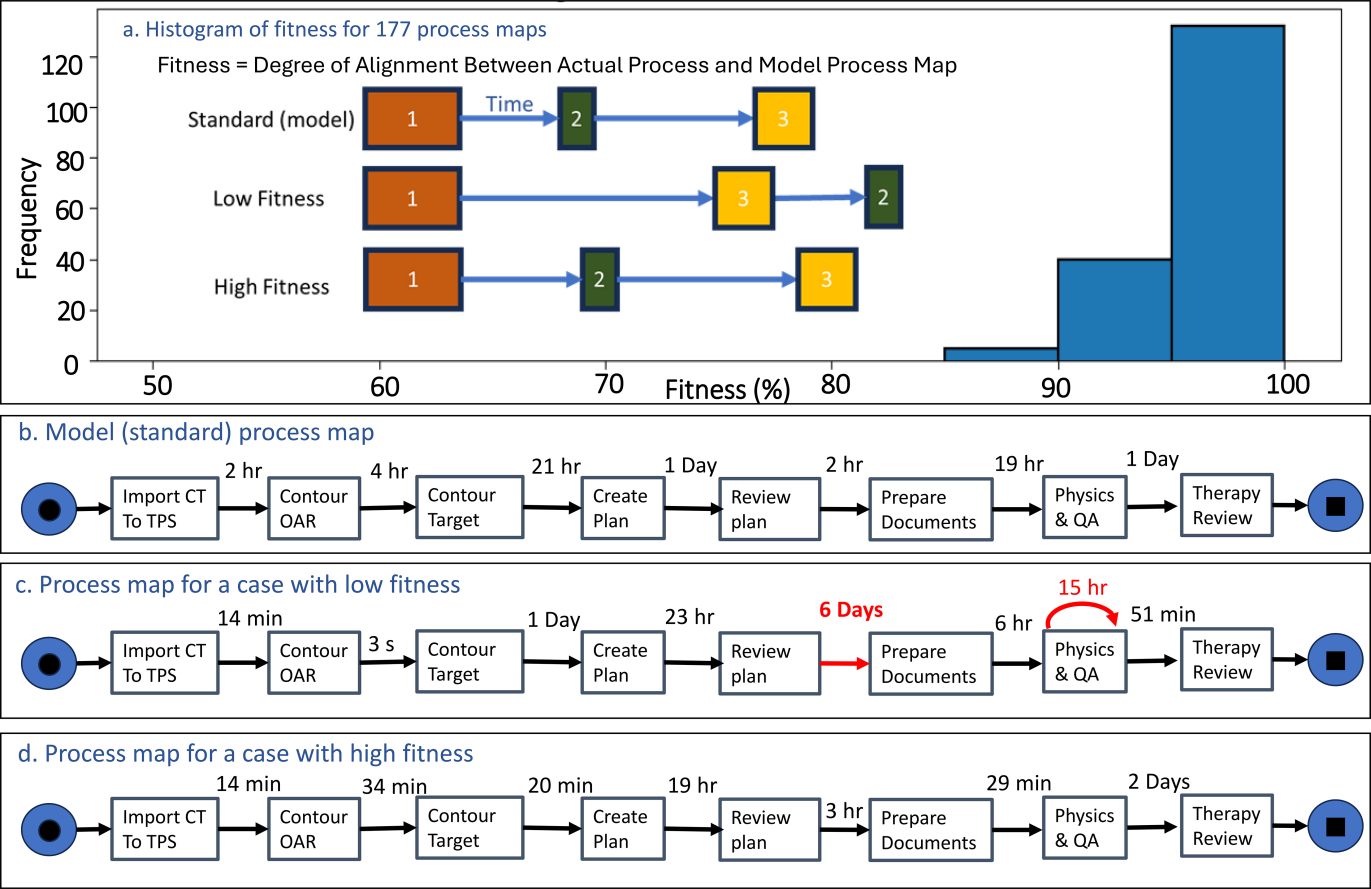
**(a)** The conformality or fitness score of different patients' process maps compared to the model process map. The histogram displays the distribution of fitness values of various processes, ranging from low to high conformity. Scores near 100% indicate near-complete conformity, akin to a “non-complex” system, where everything follows a predictable, uniform pattern. Lower scores reveal greater deviations and variability, reflecting the complex or emergent behaviors more typical of a real-world radiation oncology department. **(b)** shows the model (standard) process map, while **(c)** and **(d)** illustrate examples of process maps with low and high fitness, respectively.

The process map across 40 distinct cases was compared with a model process map. The evaluation of process conformity of patients within a certain period within the radiation oncology department is visualized in **Figure 6A**, which depicts a histogram of case-level fitness values. These values represent the degree of alignment between individual patient process maps and the department’s model process map, as described in the methods section of this manuscript.

As **Figure 6A** illustrates, there is a significant concentration of cases in the moderate to high conformity ranges, with the most frequent occurrence falling within the 60% fitness category.

The distribution also reveals the presence of cases with both lower and higher conformity, reflecting the natural variation inherent in clinical practice. Scores close to 100% represent near-complete conformity to the model process (**Figures 6B–D**), indicating minimal deviations from the standard model.

The existence of multiple variants underscores the department’s proactive stance in ensuring that patient care is not compromised by rigid adherence to protocol. The ability to self-organize and adapt treatment processes as the situation demands is crucial for delivering high-quality, safe patient care. This flexibility within the team facilitates appropriate actions, creating new variants organically, which aligns with the emergent properties of adaptability and resilience in complex systems. Application of the Plan Do Study Act (PDSA) cycle would incrementally improve protocols according to observed data, as long as the process is not tampered with (38). While adapting treatment to each individual patient introduces a level of variability that may appear chaotic, it is crucial to recognize that this flexibility is a fundamental aspect of managing complex systems in radiation oncology. Standardization can indeed help optimize resource allocation, allowing for a more focused application of adaptive care where it is most needed, thereby harmonizing the principles of adaptability and the efficient use of resources.

### 3.4 Self-organizing

**Figure 7:**
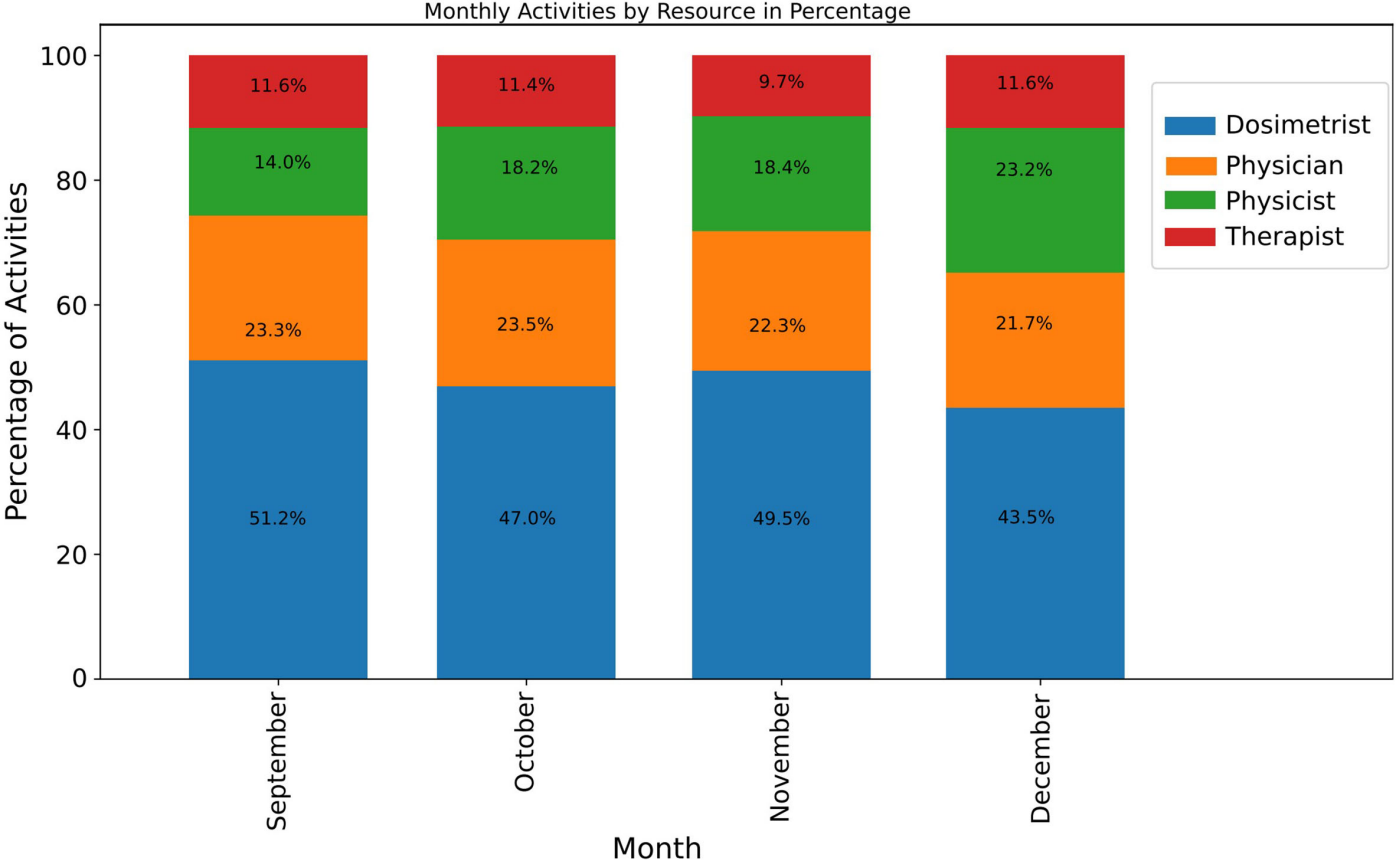
Adaptability in RO.

The results of the analysis of the distribution of activities within the radiation oncology department are depicted in **Figure 7**. As seen, there is a trend of task redistribution among the various professional roles over the monitored period. This trend suggests a characteristic self-organizing process within the department, where roles and responsibilities are dynamically adjusted in response to changing operational demands.

**Figure 7** illustrates a decrease in activities performed by dosimetrists, from 51% in September to 43% in December. Conversely, physicists’ involvement shows a progressive increase in their share of activities, culminating at 23% by year-end. Throughout the period, physician activities exhibit minor fluctuations but generally maintain a consistent quarter-share of the total activities. Here, the trend does not align with our interests since adaptability depends on the circumstances. Therapist involvement also demonstrates variability, though to a lesser extent, indicating a degree of flexibility in their operational capacity.

The emergent pattern from 7 indicates that the department is not static. It exhibits a responsive and adaptable allocation of resources, characteristic of a self-organizing system. This internal capability to adjust and realign professional activities, seemingly without explicit directive, indicates a system inherently equipped to maintain equilibrium and efficiency in the face of fluctuating healthcare landscapes. The observed shifts in task distribution demonstrate the system’s resilience, as it adapts to changing workloads and maintains functionality by reallocating responsibilities among different roles.

**Figure 8:**
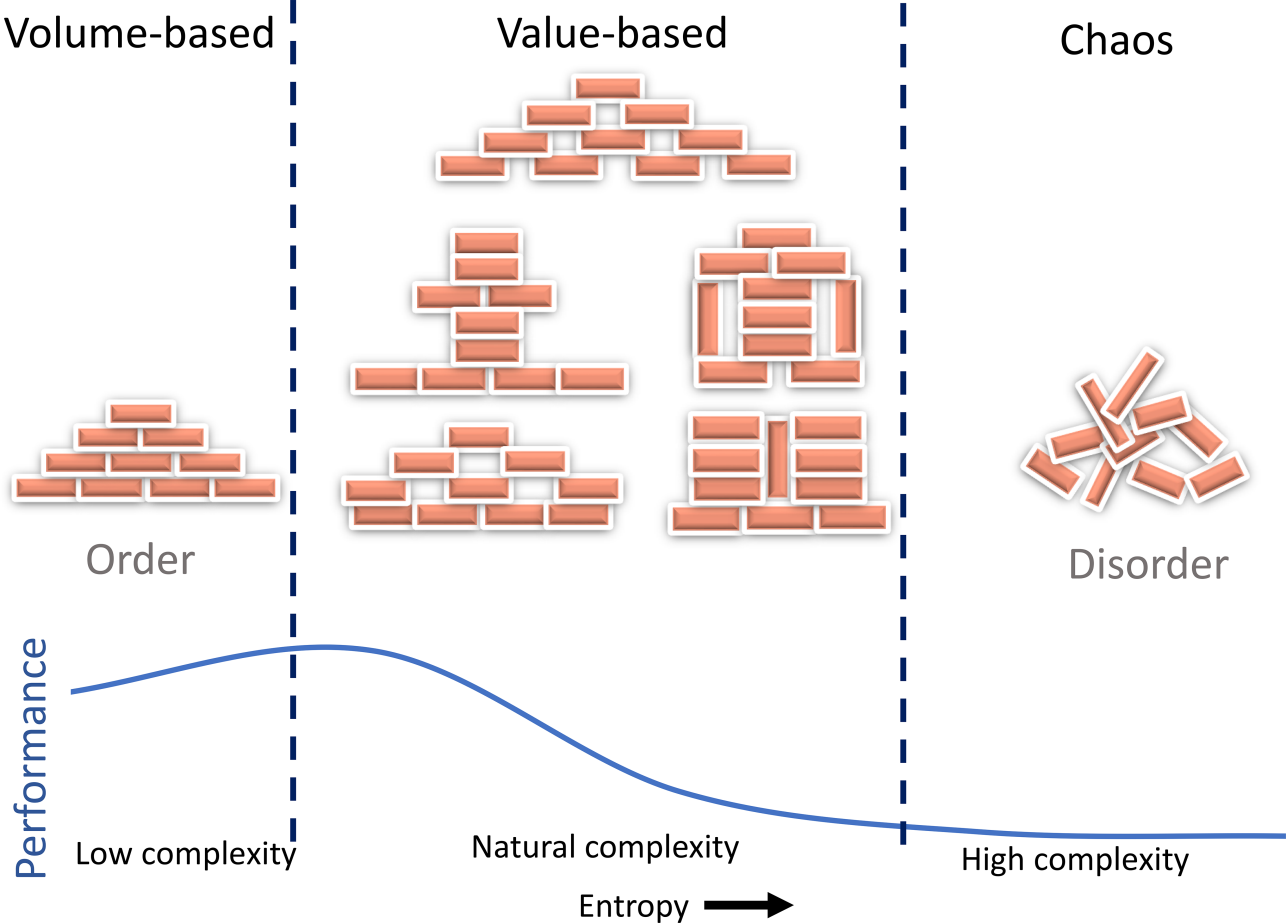
Overall, all interpretations of the results. A natural complexity in an RO department leads to the emergence of safety and quality.

**Figure 8** further synthesizes these findings by situating the department’s dynamics within broader concepts of order, disorder, chaos, and complexity. The department demonstrates an adaptive capability that aligns it with a complex system capable of balancing predictability (order) with flexibility (autonomy). This adaptability allows the system to respond effectively to evolving challenges, utilizing feedback loops and decentralized decision-making to navigate shifts in workload and resource demands. By maintaining this balance, the system exhibits resilience and efficiency, reinforcing its ability to thrive within the complex and unpredictable landscape of modern healthcare.

**Figure 8** provides an integrated overview of the findings described above, summarizing the relationship between complexity, adaptability, and system dynamics in radiation oncology. Order refers to a system that is well-organized, predictable, and stable. An example of an ordered system in healthcare is a hospital with clear protocols and procedures and a strong hierarchy of authority. Disorder refers to a system that is less organized, predictable, and stable than an ordered system. In healthcare, an example of a disordered system would be a hospital with inadequate resources, poor communication, and a lack of clear protocols and procedures. Chaos is a highly disordered and unpredictable system with little or no apparent structure. In RO, an example of a chaotic system would be a particular CT simulation that needs advanced preparation, such as patient dependency on special accessories, special setup, monitoring, etc., which was not discussed in the huddle. The culture and cultural assessments of the departments are essential hidden components of a system (39). This also requires decentralization, as in an adaptive system, decision-making is often distributed among multiple agents or sub-systems rather than being centralized (5). According to **Figure 2**, the complexity of RO systems may have surpassed a level that a single human can intellectually manage. Adaptability allows the system to respond more quickly and effectively to changing conditions to maintain safety. Cognitive diversity among agents or sub-systems with distinctive characteristics or behaviors can increase the system’s resilience and ability to respond to changing conditions (40) and the pre-requirement for this is undoubtedly the psychological safety (41). It is important to note that these terms are not mutually exclusive, and a healthcare system can have elements of order, disorder, and chaos at the same time. Understanding the dynamics and predictability of these various levels of organization can help healthcare providers and administrators improve the quality of care and the overall efficiency of the healthcare system. It is crucial to note that the balance between standardization and autonomy plays a key role in managing entropy. Standardization, protocols, policies, procedures, regulations, and guidelines reduce entropy by creating order and predictability, while autonomy and trust in employees to make decisions when needed increase entropy. This dynamic balance depends on time and space, so steering through feedback loops of errors, incident learning systems, and corrective actions is vital. Nevertheless, increasing entropy is inevitable when adapting to changes in the environment. **Figure 6A** highlights key insights, demonstrating the trade-offs between performance pressure, error rates, and patient satisfaction in the treatment planning process. From a physics, mathematics, or statistical point of view, cases with less than 100% fitness should indicate errors. However, these errors have not led to harm to patients but led to safety. Therefore, the misconception of preventing error in the field might need to be corrected to zero harm (42–44). Instead, we welcome errors (11, 45).

For a concise overview of our main findings and their implications, **Table 1** summarizes the key results presented in this study.

**Table 1:** Summary of key results and implications.

CAS property / measure	Data / model result (figure reference)	Implications
Spatiotemporal Dynamics	**Figures 1A, B** & **2**: FDA data on approved RO devices over several decades; exponential increase in RO publications. Shannon Entropy was used as the metric for complexity.	Indicates that RO complexity has escalated over time, underscoring the need for adaptive management to handle the increasing unpredictability.
Nonlinearity	**Figures 3**-**6**: Dynamic system simulations demonstrating trade-offs between pressure, performance, errors, and patient satisfaction. Increased pressure initially improves performance but eventually leads to higher errors and reduced patient satisfaction.	Highlights that nonlinear interactions can lead to unforeseen errors, emphasizing the importance of feedback loops in managing complex RO processes.
Emergence	**Figure 6**: Distribution of case-level fitness values reflecting the degree of conformity to a model process. Variations in task completion times contribute to the overall fitness score. Lower fitness often correlates with longer or more variable processing times.	Reveals that emergent patterns support self-organization in RO, though variability calls for continuous, dynamic oversight to ensure consistent quality.
Self-Organization	**Figure 7**: Shifts in activity distributions among dosimetrists, physicists, physicians, and therapists showing the reallocation of tasks over time	Demonstrates the system’s resilience via adaptive role redistribution, but also warns that increasing specialization may introduce vulnerabilities if key roles become bottlenecks.

## 4 Discussion

This study provides a quantitative demonstration of how RO functions as a complex adaptive system, extending beyond prior work that has recognized RO’s complexity but offered limited empirical proof (5, 6). By integrating Shannon Entropy, dynamic system simulations, and process mining, we identified exponential growth in network complexity, non-linear feedback loops, and self-organizing team behaviors—each underscoring the emergent nature of RO processes. Such findings contrast with linear risk management frameworks (e.g., TG-100), which can miss unforeseen failure modes in a rapidly evolving technological landscape. Our approach highlights the necessity for adaptive strategies—such as decentralized decision-making and real-time feedback loops—to effectively manage RO’s inherent nonlinearity.

### 4.1 Practical implications for radiation oncology as a complex adaptive system

Building on the quantitative findings presented in **Figures 5**-**7**, these results have direct implications for how radiation oncology departments manage safety, quality, and operational efficiency. In systems characterized by nonlinearity, feedback loops, and emergent behavior, static control strategies and linear assumptions are insufficient for sustained performance.

In complex adaptive systems, redundancy improves resilience only when it is diverse rather than duplicative. In radiation oncology, effective redundancy arises from combining heterogeneous safety mechanisms, such as inter-professional review across physicians, physicists, and dosimetrists; independent physics checks; and AI-assisted quality assurance used as a secondary safeguard rather than a replacement for human judgment. Such diversity reduces the risk of common-mode failures, particularly as treatment planning complexity and automation increase.

The nonlinear simulations illustrate how pressure, performance, errors, and patient satisfaction interact through reinforcing and balancing feedback loops (**Figure 5**). Under these conditions, centralized decision-making can delay responses to emerging risks. Decentralized structures—such as structured daily huddles, clearly defined escalation pathways, and frontline authority to pause or adapt workflows—enable faster, context-sensitive responses. These mechanisms allow local actors to correct system drift before small disturbances propagate into larger failures.

Process mining demonstrated substantial variability in workflow conformance, with many cases deviating from the model process without resulting in patient harm (**Figure 6**). In the context of a complex adaptive system, such variability often reflects necessary adaptation to patient-specific or operational constraints rather than unsafe practice. Treating all deviations as errors risks suppressing beneficial flexibility. Instead, variability should be interpreted as informative signal, allowing departments to distinguish harmful deviations from adaptive responses and to refine protocols without undermining resilience.

Finally, effective quality improvement in radiation oncology requires integrating existing tools into a feedback-driven framework. TG-100 provides structured prospective risk assessment, incident learning systems capture unanticipated events and near misses, and process mining reveals emergent workflow patterns and bottlenecks over time (**Figures 6**, **7**). When these tools operate as interconnected components rather than in isolation, they support continuous recalibration of workflows in response to evolving complexity, strengthening safety and performance in modern radiation oncology practice.

The following system-thinking approaches illustrate how these principles can be operationalized in radiation oncology practice. To manage nonlinearity, it is essential to have clear communication, shared goals, and a collective understanding of roles and responsibilities (46). Additionally, teams should be trained in problem-solving, decision-making, and conflict-resolution techniques to better navigate the unpredictable nature of healthcare systems (47).

Here, we elaborate on specific systems thinking approaches to managing the unique challenges of an RO department:

1. Risk Assessment with American Association of Medical Physicists Task Group (TG)-100: TG-100, which serves as a guideline for performing risk assessment assessments, emphasizes process mapping, providing a holistic view of the entire workflow. When failure modes are identified, their ripple effects across the system are analyzed. This ensures that corrective interventions minimize unintended consequences and avoid introducing new issues elsewhere in the system. This proactive approach aligns well with CAS principles, where interconnectedness is key.
2. Incident Learning Systems (ILS) (11): ILS represents a robust tool for managing CAS dynamics. It facilitates continuous feedback through the reporting of errors and near-misses. This iterative process enables the system to self-correct and adapt, leading to enhanced safety and reliability. By embedding ILS into daily operations, RO departments can foster a culture of learning and resilience.
3. Decentralization through Huddles and QI Meetings (46): Effective decentralization requires structured huddles and quality improvement (QI) meetings. These platforms empower teams by shifting decision-making from a central authority to collaborative groups. When implemented correctly, huddles and QI meetings enable frontline staff to address challenges in real-time, leveraging their collective expertise to adapt to evolving conditions.

It is essential to emphasize that the systems thinking approaches outlined above—TG-100, ILS, and decentralization—must not be applied in isolation. Instead, they should operate as interconnected components within a feedback-driven framework. Each intervention influences and is influenced by the others, creating a dynamic system that continually learns and adapts to maintain safety and quality in RO operations.

Although our discussion highlights universal system-thinking strategies, local context shapes how these interventions are applied. Recent reviews of precision radiation oncology similarly emphasize that increasing technological sophistication and AI-enabled workflows necessitate adaptive governance, continuous feedback, and flexible quality assurance structures rather than rigid standardization (48). A resource-rich academic center, for instance, may have the funds to invest in extensive quality assurance staff, high-end technology, and robust training programs—facilitating the rapid implementation of ILS and comprehensive TG-100 workflows. In contrast, a community-based clinic with tight budgets and limited staffing must adopt these same principles more frugally by relying on cross-functional staff performing multiple roles or external vendor support. Regulatory mandates (e.g., new accreditation standards) can further compel changes to existing workflows. By acknowledging and planning for these external factors, departments can better align system-thinking strategies with real-world constraints, ensuring that initiatives remain practical, achievable, and resilient to evolving circumstances.

The methodologies employed in this study—Shannon Entropy, dynamic system simulations, and process mining—offer valuable perspectives on the complexity of RO. Shannon Entropy highlights how adding new artifacts or information over time can increase a system’s overall complexity, while dynamic simulations underscore the nonlinear feedback loops that generate emergent behaviors. Process mining, in turn, reveals hidden workflow patterns and potential bottlenecks. Although each approach has certain inherent assumptions and limitations detailed in following subsection, Limitations and Future Directions, collectively illustrate why RO must be managed as a complex adaptive system. These methodologies underscore the importance of balancing standardization with the adaptability required to navigate the field’s rapid technological growth and diverse patient needs.

Despite these limitations, the findings underscore the importance of balancing standardization and adaptability in managing complexity, as we mentioned earlier. Policies and procedures, although required, should not overwhelm employees. Instead, they should provide “freedom within a frame,” allowing employees the autonomy to exercise their expertise effectively when needed (45). This balance ensures that while systems remain in equilibrium through standardization, they also retain the flexibility to achieve a new equilibrium in response to the dynamic environment. Feedback mechanisms such as huddles and incident learning systems (ILS) are essential for maintaining this equilibrium, enabling organizations to navigate complexity while preserving safety and quality. In practice, the optimal balance between standardization and adaptability cannot be reduced to a single static ratio. Complex adaptive systems continually evolve, meaning that what is optimal in one context may become insufficient or excessive in another. For instance, a department experiencing a surge in patient load might benefit from loosening certain protocols to accommodate urgent demands, whereas under normal conditions, tighter adherence to standardization could ensure consistent quality. Consequently, real-time feedback loops—such as huddles, incident learning systems, and cross-functional reviews—are essential for detecting shifts in workload, error patterns, and patient outcomes, and then recalibrating the standardization–adaptability balance accordingly. Rather than pinpointing a universal ‘sweet spot,’ departments should maintain the capacity to reconfigure their workflows dynamically, allowing them to remain both safe and efficient across diverse, changing circumstances.

As the radiation oncology field advances, subspecialists (e.g., dedicated dosimetrists for proton therapy, QA physicists for MR-linac, etc.) bring a high level of expertise that can reduce error rates in emerging technologies. However, these specialized roles can segment workflows, increasing the complexity of handoffs and communication chains. When critical information is confined within a single role, the broader system may become more vulnerable to disruptions if that key individual is unavailable. Moreover, highly specialized teams may find it harder to pivot or share responsibilities when patient volumes fluctuate unexpectedly. Consequently, the benefits of specialization in accuracy and efficiency must be balanced against potential drawbacks in adaptive capacity—a tension that future research could address by modeling interdisciplinary cross-training and exploring how to optimize team composition under various workflow constraints.

Recent work by Benning et al. (49) on the implementation of ICHOM (International Consortium for Health Outcomes Measurement) standard sets provides a valuable illustration of how organizations can adapt standardized outcome measures for diverse clinical environments. In evaluating 27 routinely implemented standard sets, the authors highlight how local variations—such as differing care pathways, patient populations, and resource constraints—necessitate a degree of flexibility in how these standards are applied. Simultaneously, common metrics ensure comparability and consistency in measuring outcomes across institutions. This approach supports our assertion that effective balancing does not mean a rigid, one-size-fits-all framework; rather, it involves pragmatic adaptation of core standardized practices to local realities, thus maintaining both uniform quality benchmarks and the capacity to innovate and respond to site-specific needs.

### 4.2 Comparison with other complex adaptive systems

Complex adaptive systems are encountered across a variety of high-stakes fields. For example, in aerospace engineering, safety and resilience are maintained through rigorous redundancy protocols and decentralized decision-making, as detailed by Dekker (45). These approaches ensure that even if one component fails, overall system integrity is preserved through continuous monitoring and rapid adaptive responses. Similarly, surgical teams have adopted adaptive strategies—such as structured team communication (46)—to manage the unpredictable nature of the operating room, as evidenced by the work of Lingard et al. (50). Although the specific challenges differ, both aerospace and surgical care demonstrate that a dynamic, feedback-driven approach to risk management can significantly enhance safety and performance. Radiation oncology can similarly benefit from adopting adaptive, feedback-driven risk management strategies that emphasize mechanism transfer—rather than direct imitation—from other high-reliability complex adaptive systems (14, 15).

Importantly, the relevance of these domains lies not in superficial similarity but in the transferability of underlying mechanisms. In aerospace systems, safety improvements have been achieved through the deliberate separation of monitoring and execution functions, cross-checking across cognitively independent agents, and the formalization of feedback loops that detect weak signals before they escalate into failures (15, 45). Similarly, in surgical care, structured team communication and standardized briefings have been shown to reduce communication failures, improve coordination, and enhance error detection under time pressure (46, 50). These mechanisms operate by strengthening system-level feedback, enabling rapid local adaptation, and preventing the accumulation of latent conditions—features that align directly with the properties of complex adaptive systems demonstrated in this study. In radiation oncology, analogous mechanisms include multidisciplinary plan review, decentralized escalation through huddles, and continuous feedback via incident learning systems, all of which support resilience in the presence of increasing technological and workflow complexity.

### 4.3 Limitations and future directions

Despite providing conceptual insights into complexity in RO, our methods face several constraints. First, both the Shannon Entropy and predator-prey models use simplified assumptions—treating all components as uniformly influential or omitting external factors like workforce growth, financial constraints, and regulatory shifts. Consequently, these models may underestimate the true complexity of modern RO. Second, process mining and social network analysis rely on high-quality event log data; incomplete or inconsistent records can limit the accuracy of discovered workflows. Third, our focus on sub-systems rather than the entire RO ecosystem underscores the need for broader, interdisciplinary collaborations that incorporate diverse clinical settings and larger data sets. Future work could integrate workforce dynamics, resource availability, and socio-political pressures to form more holistic models of complexity in RO. Such research may also benefit from comparing multiple departments or healthcare systems, shedding light on how various contextual factors affect the balance between standardization and adaptability. Ultimately, refining these approaches will help us better capture the emergent, adaptive nature of RO, guiding improvements in patient safety, workflow efficiency, and quality of care.

Major initiatives such as the AAPM TG-100 report (6) have advanced radiation oncology by introducing systematic risk assessment methods—most notably, Failure Modes and Effects Analysis (FMEA) and the calculation of Risk Priority Numbers (RPN). These tools are valuable for identifying and mitigating risks anticipated during the risk assessment phase. However, they operate assuming potential failure modes can be pre-defined and treated as predictable outcomes. In contrast, complex adaptive systems are characterized by non-linear interactions and emergent behaviors that may give rise to incidents never foreseen during traditional risk assessments. This limitation means that in dynamic environments like radiation oncology, unanticipated events or cascading failures can occur—events that standard FMEA might not capture. By integrating system-thinking methodologies such as Shannon Entropy, dynamic simulations, and process mining, our approach aims to provide a more comprehensive view of the inherent complexity and unpredictability in RO workflows, thereby complementing existing risk management frameworks.

Future investigations may also consider more holistic methodologies like STPA (System-Theoretic Process Analysis) (14) move beyond linear cause-effect assumptions to capture emergent behaviors and feedback loops in socio-technical systems. This approach may prove especially valuable as radiation oncology increasingly adopts artificial intelligence (AI)-based solutions for treatment planning, QA, and error detection—technologies that can introduce novel, unpredictable risks. For instance, new guidelines Hurkmans et al. (51) and quality assurance frameworks by Claessens et al. (52) highlight the promise and complexity of AI-driven tools. Merging these emerging methods with our system-thinking framework, radiation oncology can better respond to new sources of complexity, enhancing patient safety and operational resilience in an era of rapid technological change.

## 5 Conclusions

In summary, this study demonstrates that radiation oncology operates as a complex adaptive system characterized by nonlinearity, emergent behavior, and self-organization. By integrating Shannon Entropy, dynamic system simulations, and process mining, we provide quantitative evidence of how complexity in radiation oncology has increased over time and how system behavior arises from interacting feedback loops rather than linear cause–effect relationships. While prior work has conceptually acknowledged the complexity of radiation oncology, this study advances the field by empirically demonstrating multiple defining properties of complex adaptive systems and linking them to operational decision-making and risk management. As radiation oncology continues to advance, ongoing research into its intricate feedback loops will inform next-generation management frameworks, ensuring resilience and effectiveness in an ever-changing healthcare landscape.

## Data Availability

The original contributions presented in the study are included in the article/**Supplementary Material**. Further inquiries can be directed to the corresponding author.
